# Antimicrobial susceptibility of Enterococcus and Bacillus species isolated from commercial probiotic products indicated for use in cattle

**DOI:** 10.3168/jdsc.2026-1069

**Published:** 2026-07-27

**Authors:** Rúbia Branco-Lopes, Emmanuel Okello, Karen Tonooka, Hedmon Okella, Richard V.V. Pereira, Noelia Silva-del-Río

**Affiliations:** 1 University of California Agriculture and Natural Resources, Cooperative Extension, Tulare, CA 93274; 2 Veterinary Medicine Teaching and Research Center, School of Veterinary Medicine, University of California, Davis, Tulare, CA 93274; 3 Department of Population Health and Reproduction, School of Veterinary Medicine, University of California, Davis, Davis, CA 95616; 4 University of California Agriculture and Natural Resources, Cooperative Extension, Hollister, CA 95023

## Abstract

Assessment of antimicrobial resistance is an important component of probiotic safety evaluation, particularly as the use of these products increases on commercial dairy farms. Because microbial feed additives should not contribute to the dissemination of antimicrobial resistance (AMR), characterizing the antimicrobial susceptibility of microorganisms used in commercial probiotic products is critical. The objective of this study was to evaluate the phenotypic antimicrobial susceptibility of *Enterococcus* spp. and *Bacillus* spp. isolated from commercially available cattle probiotics. Cattle probiotic products labeled to contain at least 1 *Enterococcus* spp. or *Bacillus* spp. were identified through different sources. A total of 35 products were included. Phenotypic antimicrobial susceptibility of the bacterial isolates was evaluated against 16 antimicrobials using the broth microdilution method, and data were interpreted according to microbiological cut-off values. Based on 16S rRNA gene sequencing, isolates were identified as *Enterococcus faecium* (n = 15), and *Enterococcus hirae* (n = 1), *Bacillus amyloliquefaciens* (n = 1), *Bacillus licheniformis* (n = 1), *Bacillus subtilis* (n = 7), and *Bacillus velezensis* (n = 13). All *Enterococcus* isolates were susceptible to chloramphenicol, kanamycin, tetracycline, and vancomycin. Three isolates were resistant to erythromycin, 1 isolate was resistant to tylosin, and 1 isolate exhibited resistance to both erythromycin and tylosin. All 22 *Bacillus* spp. isolates were susceptible to tetracycline and vancomycin, whereas resistance was detected to chloramphenicol (n = 1) and erythromycin (n = 4). The phenotypic AMR observed in some isolates suggests a need for further investigation into the safety of commercially available probiotics, including the presence and potential transferability of AMR genes and associated mobile genetic elements.

## Graphical Abstract

**Figure:**
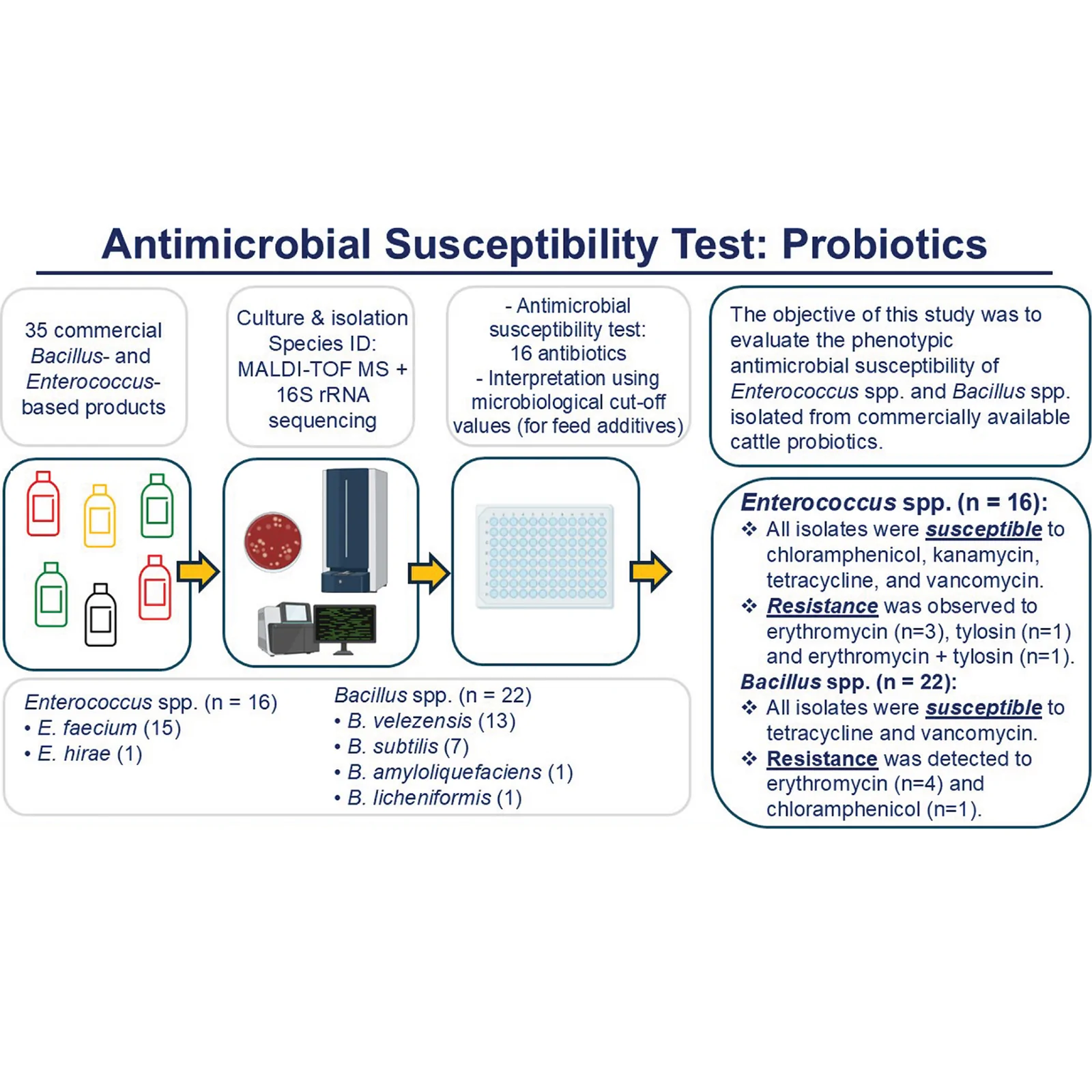

Misuse and overuse of antimicrobials is considered a contributing factor in the emergence and spread of antimicrobial resistance (**AMR**), which is a global threat to human and animal health (Davies and Davies, 2010). In dairy cattle, misdiagnosis of diseases by farm personnel can lead to inappropriate treatment decisions (Olson et al., 2019), which may contribute to AMR. In addition, management practices (e.g., feeding waste milk) have been associated with AMR in dairy cattle (Springer et al., 2019).

Probiotics, defined as “live microorganisms that, when administered in adequate amounts, confer a health benefit on the host” (Hill et al., 2014), have been investigated as an alternative to minimize the use of antimicrobials. The use of probiotics has been shown to reduce the need for therapeutic treatments and the duration of diarrhea in calves (Timmerman et al., 2005; Renaud et al., 2019), and a comparable cure rate to antimicrobials in mastitic cows (Kitching et al., 2019). In the United States, probiotic supplementation is widely adopted by dairy producers, with 38% of large dairy operations using it for heifers and 42% for cows (USDA, 2016).

Although some microorganisms used as probiotics are generally recognized as safe (**GRAS**) or have qualified presumption of safety (**QPS**) status, research interest in assessing their safety is growing (Merenstein et al., 2023). One of the concerns associated with probiotic use is the presence of AMR genes and the potential transfer of these genes to pathogenic bacteria (Li et al., 2020). Additionally, an in vivo study suggested that antimicrobial resistant fecal coliform counts were higher after probiotic supplementation in calves (*Bacillus* and *Lactobacillus* spp.; Corbett et al., 2015). Resistance in probiotic bacteria can be intrinsic or acquired and only the latter poses a risk of dissemination through horizontal gene transfer (Li et al., 2020). Phenotypic antimicrobial susceptibility testing provides a valuable first step to understand resistance profiles, though it does not fully address the transferability of resistance traits (EFSA FEEDAP Panel et al., 2018).

*Enterococcus* and *Bacillus* spp. are commonly investigated probiotics for dairy cattle use (Branco-Lopes et al., 2023). However, limited information exists on the antibiotic resistance or susceptibility of *Enterococcus*- or *Bacillus*-based probiotics marketed for use in cattle. A prior study examined the AMR of commercial probiotic strains of *E. faecium* for cattle and swine, finding that some (15 out of 22) isolates were resistant to medically important antimicrobials (Amachawadi et al., 2018). Given the possible role of probiotics in antimicrobial stewardship programs and their extensive use as feed additives by dairy producers, it is important to assess their safety. Microbial feed additives should not contribute to the dissemination of AMR; therefore, characterizing the antimicrobial susceptibility profiles of microorganisms used in commercial probiotic products is a critical component of their safety assessment (EFSA FEEDAP Panel et al., 2018). Thus, the objective of this study was to evaluate the phenotypic antimicrobial susceptibility of *Enterococcus* spp. and *Bacillus* spp. isolated from commercially available cattle probiotics.

Cattle probiotic products labeled to contain at least one *Enterococcus* spp. or *Bacillus* spp. were identified from compendia, regulatory registries, expert consultation, and targeted internet searches (Bayer HealthCare, 2010; Feedstuffs, 2019; European Commission, 2022; Health Canada, 2022). Thirty-five products were included and coded for confidentiality (15 with *Enterococcus* spp. only [14 *E. faecium* and 1 *E. lactis* + *E. faecium*], 9 with *Bacillus* spp. only [5 *B. subtilis*, 2 *B. licheniformis* + *B. subtilis*, 1 *B. amyloliquefaciens*, and 1 for which species was not reported, only genus], and 11 with both genera [7 *E. faecium* + *B. subtilis*, 3 *E. faecium* + *B. subtilis* + *B. licheniformis*, and 1 *E. faecium* + *B. subtilis* + *B. licheniformis* + *Heyndrickxia coagulans*; formerly *B. coagulans*]). When multiple target bacteria within the genera of interest were listed on the product label, separate isolations were attempted for each. For each product, 0.5 g or 0.5 mL was reactivated in 5 mL of tryptic soy broth (**TSB**) at 35°C for 2 h. For *Enterococcus* spp., 0.5 mL of the culture was plated onto bile esculin agar (**BEA**) and incubated at 35°C for 24 h. To ensure purity, the putative colonies were streaked onto BEA and subsequently onto blood agar (Remel, Lenexa, KS), with both plates being incubated at 35°C for 24 h each time. For *Bacillus* spp., culture was plated onto blood agar and incubated at 37°C for 18 h. For purification, the putative colonies were streaked onto blood agar plates and incubated at 37°C for 18 h. Presumptive isolates were stored at −80°C in TSB containing 20% glycerol (vol/vol).

For identification, isolates were recovered on blood agar (35°C for 24 h and 37°C for 18 h for *Enterococcus* spp. and *Bacillus* spp. respectively) and species identification was performed by MALD-TOF MS at California Animal Health and Food Safety Laboratory System (Tulare, CA) and by 16S rRNA gene sequencing. However, 16S rRNA gene sequencing may not reliably differentiate closely related species and does not discriminate at strain level. Two isolates identified as *Pantoea* spp. and *Acinetobacter baumannii* by MALDI-TOF-MS were excluded from further analyses. For sequencing, genomic DNA was extracted using commercial kits (DNeasy Blood and Tissue Kit for *Enterococcus* spp.; PureLink Microbiome Kit for *Bacillus* spp.), and DNA concentration and purity were assessed by spectrophotometry. The 16S rRNA gene was amplified using primers 27F (5′-AGAGTTTGATCMTGGCTCAG-3′) and 1492R (5′-TACGGTTACCTTGTTACGAC-3′). The PCR amplicons were sequenced at University of California, Berkeley (DNA Sequencing Facility, Berkeley, CA). Sequencing reads were analyzed using the CLC Genomics Workbench v24.0 (Qiagen, Hilden, Germany). Paired-end reads were trimmed and de novo assembled into consensus sequences, which were compared against the National Center for Biotechnology Information (**NCBI**) nucleotide collection database using NCBI's standard nucleotide basic local alignment search tool (BLASTn; https://blast.ncbi.nlm.nih.gov/Blast.cgi?PROGRAM=blastn&BLAST_SPEC=GeoBlast&PAGE_TYPE=BlastSearch). The sequences were deposited in the GenBank database (https://www.ncbi.nlm.nih.gov/genbank/about/; accession numbers PV569856–PV569893).

Antimicrobial susceptibility testing (**AST**) of the probiotic isolates was performed by the broth microdilution method in accordance with the Clinical and Laboratory Standards Institute (CLSI, 2018) to determine the MIC of antimicrobials. Bacterial suspensions were prepared to an equivalent 0.5 McFarland turbidity standard by mixing individual colonies with demineralized water (Trek Diagnostics Systems, Cleveland, OH). For *Bacillus* isolates, the suspension was prepared after vortexing bacterial colonies with glass beads for 2 min. A 10-μL aliquot of the standardized bacterial suspension was added to Mueller–Hinton broth (Trek Diagnostics Systems, Cleveland, OH) and vortexed. A total of 50 μL of the final inoculum suspension were dispensed into each well of the gram-positive National Antimicrobial Resistance Monitoring System (**NARMS**) panel plates (CMV3AGPF, Trek Diagnostics Systems, Cleveland, OH) using the Sensititer automated inoculation delivery system (Trek Diagnostics Systems, Cleveland, OH). This panel plate was developed for AMR surveillance of *Enterococcus* as part of NARMS, which monitors *Enterococcus* isolates from food-producing animals and retail meats. It includes antimicrobials relevant to both human and veterinary medicine and aligns with European Food Safety Authority (**EFSA**) guidance for evaluating the antimicrobial susceptibility of microorganisms intended for use in animal nutrition (EFSA FEEDAP Panel et al., 2018). Plates were incubated for 24 h at 35°C and read and reported using the Sensititre Vizion Digital MIC Viewing System (model 436-A, Thermo Fisher Scientific, Waltham, MA) and the Thermo Scientific Sensititre SWIN Software System, version 3.3 (3.3.2.7). The present study was conducted within the context of the safety assessment of microbial feed additives, specifically the evaluation of AMR. Therefore, antimicrobial susceptibility phenotypes of isolates from commercial probiotics were interpreted using EFSA microbiological cut-off values (EFSA FEEDAP Panel et al., 2018). These cut-off values were developed specifically for microorganisms used in animal nutrition and are intended to assess the potential presence of acquired AMR. *Enterococcus faecalis* ATCC 29212 was used as the quality control strain and was tested on 1 plate each day of AST. The MIC value at which ≥50% (**MIC_50_**) and ≥90% (**MIC_90_**) of the isolates in a test population are inhibited, respectively, were calculated according to Schwarz et al. (2010). For each antimicrobial with an available EFSA microbiological cut-off value, the percentage of resistant isolates was calculated as the number of resistant isolates divided by the total number of isolates tested, multiplied by 100.

Bacterial growth was observed in all products except for 6 *Enterococcus*-based products (labeled as *E. faecium*), which showed no visible growth despite repeated culture attempts. In addition, 4 products labeled as containing both *Enterococcus* and *Bacillus* yielded only *Bacillus* isolates. The probiotic definition is linked to microbial viability (Hill et al., 2014). However, the inability to culture probiotic bacteria does not necessarily equate to a loss of microbial viability because bacteria could shift and remain metabolically active but not culturable (Davis, 2014). Species identification based on MALDI-TOF-MS followed by 16S rRNA gene sequencing identified *Enterococcus* isolates as *E. faecium* (n = 15), and *E. hirae* (n = 1) and *Bacillus* isolates as *B. amyloliquefaciens* (n = 1), *B. licheniformis* (n = 1), *B. subtilis* (n = 7), and *B. velezensis* (n = 13).

The MIC of antimicrobials for *Enterococcus* spp. are presented in Table 1. All isolates were susceptible to chloramphenicol, kanamycin, tetracycline, and vancomycin. Three isolates (18.75%) were resistant to erythromycin, 1 isolate (6.25%) was resistant to tylosin, and 1 isolate (6.25%) was resistant to both erythromycin and tylosin. Although gentamicin susceptibility was not interpreted because the EFSA microbiological cut-off value (>32 µg/mL) was not encompassed by the concentration range included on the NARMS plate (128–1,024 µg/mL), 1 isolate exhibited a high MIC value of 1,024 µg/mL, consistent with a resistant phenotype. The MIC of antimicrobials for *Bacillus* spp. are presented in Table 2. All isolates showed susceptibility to both tetracycline and vancomycin. Resistance to chloramphenicol (n = 1; 4.55%) and erythromycin (n = 4; 18.18%) was observed.

**Table 1:** Distribution of the MIC, MIC_50_, and MIC_90_ values of 16 antimicrobials among 16 *Enterococcus* spp. isolates^1^

Antimicrobial	MIC value (μg/mL)																				
	0.015	0.03	0.06	0.12	0.25	0.5	1	2	4	8	16	32	64	128	256	512	1,024	2,048	RI^2^ (%)	MIC_50_	MIC_90_
Chloramphenicol										*16*									0	8	8
Ciprofloxacin							9	2	5										—	1	4
Daptomycin								1	12	3									—	4	8
Erythromycin									*12*	**4**									25.00	4	8
Gentamicin														15			1		—	128	128
Kanamycin														*7*	*6*	*3*			0	256	512
Lincomycin							5			11									—	8	8
Linezolid								15	1										—	2	2
Nitrofurantoin													16						—	64	64
Penicillin								5	8	1	2								—	4	16
Quinupristin/ dalfopristin						4		11	1										—	2	2
Streptomycin																16			—	512	512
Tetracycline							*16*												0	1	1
Tigecycline			2	14															—	0.12	0.12
Tylosin								*8*	*6*	**2**									12.50	2	8
Vancomycin						*12*	*4*												0	0.5	1

1 The different typefaces indicate the antimicrobial susceptibility phenotype; italic represents susceptible and bold indicates resistant based on microbiological cut-off values defined by EFSA (EFSA FEEDAP Panel et al., 2018). Regular (nonbold, nonitalic) type numbers indicate MIC values for which cut-off values were unavailable or not applicable. Blank spaces indicate that no isolates had that MIC value or that the concentration was not evaluated. Cut-off values (μg/mL): chloramphenicol (>16), erythromycin (>4), kanamycin (>1,024), tetracycline (>4), tylosin (>4), and vancomycin (>4); isolates above these values were considered resistant. Although cut-off values are available for gentamicin and streptomycin, they were not interpreted due to the high-level range of the plate.

2 RI = % of resistant isolates (the number of resistant isolates divided by the total number of isolates tested, multiplied by 100).

**Table 2:** Distribution of the MIC, MIC_50_, and MIC_90_ values of 16 antimicrobials among the 22 *Bacillus* spp. isolates^1^

Antimicrobial	MIC value (μg/mL)																				
	0.015	0.03	0.06	0.12	0.25	0.5	1	2	4	8	16	32	64	128	256	512	1,024	2,048	RI^2^ (%)	MIC_50_	MIC_90_
Chloramphenicol								*10*	*7*	*4*	**1**								4.55	4	8
Ciprofloxacin				20		1	1												—	0.12	0.12
Daptomycin					6	10	2		4										—	0.5	4
Erythromycin					*17*	*1*				**4**									18.18	0.25	8
Gentamicin														22					—	128	128
Kanamycin														22					—	128	128
Lincomycin										22									—	8	8
Linezolid						16	4		2										—	0.5	1
Nitrofurantoin										4	16		2						—	16	16
Penicillin					19		1	1	1										—	0.25	1
Quinupristin/ dalfopristin							1	17	4										—	2	4
Streptomycin																22			—	512	512
Tetracycline							*15*	*3*	*2*	*2*									0	1	4
Tigecycline		1	2	15	4														—	0.12	0.25
Tylosin					3	14	2	1	2										—	0.5	2
Vancomycin					*19*	*2*	*1*												0	0.25	0.5

1 The different typefaces indicate the antimicrobial susceptibility phenotype; italic represents susceptible and bold indicates resistant based on microbiological cut-off values defined by EFSA (EFSA FEEDAP Panel et al., 2018). Regular (nonbold, nonitalic) type numbers indicate MIC values for which cut-off values were unavailable or not applicable. Blank spaces indicate that no isolates had that MIC value or that the concentration was not evaluated. Cut-off values (μg/mL): chloramphenicol (>8), erythromycin (>4), tetracycline (>8), and vancomycin (>4); isolates above these values were considered resistant. Although cut-off values are available for gentamicin, kanamycin and streptomycin, they were not interpreted due to the high-level range of the plate.

2 RI = % of resistant isolates (the number of resistant isolates divided by the total number of isolates tested, multiplied by 100).

The present study was conducted within the context of the safety assessment of microbial feed additives, specifically the evaluation of AMR. Antimicrobial susceptibility testing is used as a tool to assess the safety of probiotic products under different guidelines. The FAO/WHO developed a guideline for the evaluation of probiotics, which highlighted the need for developing a standardized AST for microorganisms used as probiotics (FAO/WHO, 2002), particularly to establish breakpoints distinguishing susceptible from resistant strains. More recently, the EFSA Panel on Additives and Products or Substances used in Animal Feed (**FEEDAP**) developed a guidance document to assist applications for marketing probiotics and defined microbiological cut-off for 13 relevant antimicrobials (EFSA FEEDAP Panel et al., 2018). These cut-off values were used to interpret the antimicrobial susceptibility phenotypes observed in the present study. Additionally, AMR testing is required as part of the safety assessments of probiotics by regulatory agencies in several countries (Roe et al., 2022).

The EFSA established the QPS approach for live microorganisms used in foods and feeds (EFSA, 2026). Although safety assessments are conducted at the strain level, the extent of the assessment depends on the QPS status of the species. *Enterococcus faecium* lacks QPS status because of the presence of potential harmful traits and requires a full safety assessment (EFSA, 2026). In contrast, all *Bacillus* species identified in this study (*B. subtilis*, *B. licheniformis*, *B. amyloliquefaciens*, and *B. velezensis*) have QPS status and are subject to a simplified safety assessment (EFSA, 2026). However, AMR is assessed in both cases. Under the QPS framework, acquired AMR genes associated with an AMR phenotype are considered a safety concern. The FDA has not issued a list of animal food GRAS probiotic species; however, they provide information on GRAS notices, detailing the safety assessments conducted. Currently, GRAS notices exist for specific strains of *B. subtilis* and *B. velezensis*, whereas no GRAS notifications were identified for *Enterococcus* spp., *B. licheniformis*, or *B. amyloliquefaciens* in the FDA inventory. Our findings support the importance of safety assessment because phenotypic resistance was observed in both *Enterococcus* and *Bacillus* isolates.

In agreement with our results, resistance to erythromycin (MIC ≥ 8 μg/mL) and chloramphenicol (MIC ≥ 16 μg/mL) has been reported in *Bacillus* spp. isolated from probiotics for food animals (Tran et al., 2024). Previous research suggests that resistance to both antimicrobials may be intrinsic in certain *Bacillus* species, including *B. licheniformis* and *B. paralicheniformis* (Agersø et al., 2019). Intrinsic resistance is independent of prior antimicrobial exposure and is associated with chromosomal genes that are not transferable through horizontal gene transfer (EFSA FEEDAP Panel et al., 2018). Consequently, intrinsic resistance is not considered a safety concern by the FEEDAP Panel. However, because phenotypic susceptibility testing alone cannot distinguish between intrinsic and acquired resistance, genomic characterization is necessary to determine the relevance of the resistance phenotypes observed in this study.

In the present study, resistance to macrolides (erythromycin and tylosin) was observed among *Enterococcus* isolates. Macrolides are considered critically important antimicrobials in both human and veterinary medicine. In line with our results, *E. faecium* isolated from cattle probiotics showed resistance to erythromycin (MIC ≥ 8 μg/mL; Shridhar et al., 2022). The resistant strain carried the macrolide-associated gene *msrC*. Interestingly, *msrC* was identified in all strains evaluated in that study, including those that were phenotypically susceptible to erythromycin. In addition, Deng et al. (2021) reported erythromycin resistance (MIC ≥ 8 μg/mL) in 97% of *Enterococcus* isolates recovered from commercial probiotic products intended for human, animal, aquaculture, or plant use.

In contrast to our findings, Amachawadi et al. (2018) reported no tylosin resistant *E. faecium* isolates from commercial cattle and swine probiotic products. However, their study applied a breakpoint of ≥32 μg/mL for tylosin, which is higher than the EFSA microbiological cut-off of 4 μg/mL for *E. faecium* (EFSA FEEDAP Panel et al., 2018). In their study, 2 strains from cattle probiotics had MIC values of 8 μg/mL, suggesting that resistant isolates may also have been present when evaluated using EFSA microbiological cut-off values.

In our study, all isolates were susceptible to vancomycin and tetracycline. Vancomycin susceptibility holds crucial importance because *E. faecium* is a major species involved in vancomycin-resistant enterococci nosocomial infections (O'Toole et al., 2023). Susceptibility to tetracycline is also relevant because tetracyclines are commonly used in dairy cattle production (Abdelfattah et al., 2021). Our findings for vancomycin susceptibility in both *Enterococcus* and *Bacillus* probiotics were consistent with previous studies reporting susceptibility in *E. lactis* JDM1 (MIC ≤ 0.5 µg/mL; Fu et al., 2022), *Enterococcus* spp. isolated from commercial livestock probiotics (MIC = 0.5–2 µg/mL; Amachawadi et al., 2018), and *Bacillus* spp. isolated from probiotics for food animals (MIC = 0.25–4 µg/mL; Tran et al., 2024). In a previous study evaluating probiotics for food animals, all *Enterococcus* isolates were susceptible to tetracycline (MIC ≤ 0.125 µg/mL), whereas tetracycline resistance was detected in some *Bacillus* isolates (MIC ≥ 16 μg/mL; Tran et al., 2024).

Although valuable, phenotypic data does not address the presence of AMR genes, which are critical for understanding the potential for horizontal gene transfer. The inability to culture bacteria from some probiotic products raises questions about their viability. Additionally, MALDI-TOF MS and 16S rRNA gene sequencing may not reliably distinguish *B. licheniformis* from *B. paralicheniformis*. This distinction may be relevant for safety assessment because, although both species have QPS status, *B. paralicheniformis* is subject to the additional qualification of absence of bacitracin production ability (EFSA BIOHAZ Panel et al., 2023). These limitations highlight areas for future research to enhance the understanding and safety assessment of probiotics in cattle.

Phenotypic resistance observed in some isolates highlights the need for further studies to evaluate the presence and potential transferability of AMR genes and associated mobile genetic elements. These evaluations are important components of the safety assessment of probiotic microorganisms used as feed additives.
